# Novel keyword co-occurrence network-based methods to foster systematic reviews of scientific literature

**DOI:** 10.1371/journal.pone.0172778

**Published:** 2017-03-22

**Authors:** Srinivasan Radhakrishnan, Serkan Erbis, Jacqueline A. Isaacs, Sagar Kamarthi

**Affiliations:** Department of Mechanical and Industrial Engineering, Northeastern University, Boston, Massachusetts, United States of America; Universidad Nacional de Mar del Plata, ARGENTINA

## Abstract

Systematic reviews of scientific literature are important for mapping the existing state of research and highlighting further growth channels in a field of study, but systematic reviews are inherently tedious, time consuming, and manual in nature. In recent years, keyword co-occurrence networks (KCNs) are exploited for knowledge mapping. In a KCN, each keyword is represented as a node and each co-occurrence of a pair of words is represented as a link. The number of times that a pair of words co-occurs in multiple articles constitutes the weight of the link connecting the pair. The network constructed in this manner represents cumulative knowledge of a domain and helps to uncover meaningful knowledge components and insights based on the patterns and strength of links between keywords that appear in the literature. In this work, we propose a KCN-based approach that can be implemented prior to undertaking a systematic review to guide and accelerate the review process. The novelty of this method lies in the new metrics used for statistical analysis of a KCN that differ from those typically used for KCN analysis. The approach is demonstrated through its application to nano-related Environmental, Health, and Safety (EHS) risk literature. The KCN approach identified the knowledge components, knowledge structure, and research trends that match with those discovered through a traditional systematic review of the nanoEHS field. Because KCN-based analyses can be conducted more quickly to explore a vast amount of literature, this method can provide a knowledge map and insights prior to undertaking a rigorous traditional systematic review. This two-step approach can significantly reduce the effort and time required for a traditional systematic literature review. The proposed KCN-based pre-systematic review method is universal. It can be applied to any scientific field of study to prepare a knowledge map.

## 1. Introduction

The structure of scientific/technical knowledge is most commonly explored using two network-based methods: co-citation and keyword co-occurrence networks [1–5]. While a co-citation network focuses on studying the structure of scientific communication by analyzing links between citations in the literature, a keyword co-occurrence network (KCN) focuses on understanding the knowledge components and knowledge structure of a scientific/technical field by examining the links between keywords in the literature. The present work focuses on the analysis methods based on KCNs, which have been used in theoretical and empirical studies to explore research topics and their relationships in select scientific fields [4–12]. These studies have demonstrated practical value and advantages of KCN-based analysis over traditional literature review approaches [1].

**Fig 1:**
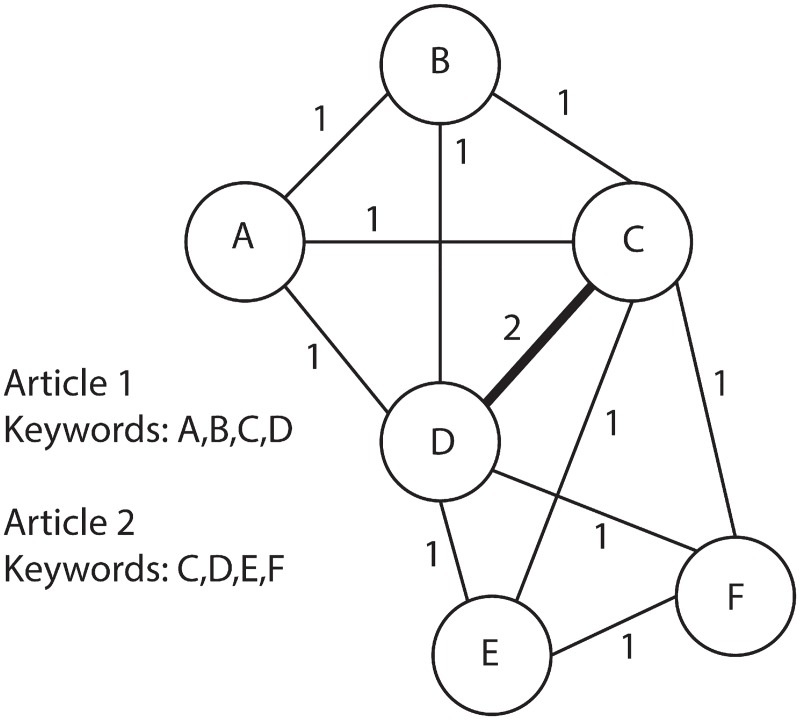
Example of a simple Keyword Co-occurrence Network (KCN). The nodes indicate the keywords published in journal articles and the links represent the co-occurrence of the words; the numbers on the links indicate the weights with the thickness of the links shown proportionally to their weight.

A KCN is created by treating each keyword as a node and each co-occurrence of a pair of words as a link between those two words (see Fig 1). The number of times that a pair of words co-occurs constitutes the weight of the link connecting these two keywords. The network constructed in this manner represents a weighted network.

A few studies have explored keyword co-occurrence (or co-citation networks) as weighted networks [1, 13–16]. However the metrics used to analyze the topographical structure of a network are generally limited to two measures: *betweeness centrality* and *modularity*. Betweenness centrality of a node captures the number of times the node is included in the shortest paths between all pairs of nodes in the keyword network. On the other hand, modularity represents the ability of the network to decompose into meaningful modules. In this work, the authors investigate several other analyses techniques including the study of average weight as a function of end point degree, average weighted nearest neighbor's degree as a function of degree, weighted clustering coefficient as a function of degree, and strength as a function of node degree. In addition, the authors introduce a visual analysis and a chronological analysis (as explained in visual analysis section) to overcome the biases of statistical analysis towards topical keywords and to study the evolution of network characteristics over time.

The proposed KCN-based analyses are evaluated using the nano- Environmental, Health, and Safety (nanoEHS) risk literature. This literature is selected for application of the technique, because 1) the nanoEHS risk field has emerged over the past decade, and 2) a detailed literature review of this field is available to validate the KCN-based observations and conclusions. Erbis et al., [17] have conducted a systematic review of nanoEHS risk literature using the traditional manual approach. We consider their findings to validate the results obtained from KCN-based analyses.

### 1.1 Theory and application

The proliferation of information in World Wide Web is accompanied by information classification and categorization issues. A user-driven categorization of information has given rise to a popular trend called as Collaborative Tagging (or Folksonomy), which allows users to categorize information using tags. The tags are keywords that facilitate information search and retrieval. Traditional classification methods, unlike collaborative tagging methods, are guided by domain experts. Jacob [18] clearly explains the difference between categorization and classification. He states that “Categorization divides the world of experience into groups or categories whose members share some perceptible similarity within a given context. That this context may vary and with it the composition of the category which is the very basis for both the flexibility and the power of cognitive categorization.” In contrast, according to Jacob [18], “Classification as process involves the orderly and systematic assignment of each entity to one and only one class within a system of mutually exclusive and non-overlapping classes; it mandates consistent application of these principles within the framework of a prescribed ordering of reality.” Supporters of tagging argue that a classification scheme is futile if the users cannot understand what the experts have defined [19, 20]. Proponents of classification point out that tagging schemes suffer from several issues including ambiguity in the meaning of tags, proliferation of synonyms that create informational redundancy, and incursion of personal utility in tagging process [19]. These limitations may cause disintegration of information into several meaningless silos. Focusing on tags as basic dynamical entities, the process of collaborative tagging falls within the scope of semiotic dynamics [21–23], a new field that studies how populations of humans or agents can establish and share semiotic systems (i.e., systems of “signs” or symbols) driven by their use in communication or information management. Folksonomies exhibit dynamical aspects similar to the ones observed in human languages such as the establishment of naming conventions, competition between terms, and takeovers by neologisms [23, 24]. It is interesting to note that the keyword selection process in scientific literature is a combination of classification and tagging schemes. Editors propose a set of thematic keywords to classify research work submitted for review and publication of articles and at the same time, authors propose a set of keywords that they think best represents their research work. The keywords appearing in research articles serve search and retrieval functions. Earlier studies report [25] that a KCN-based analysis can provide meaningful knowledge patterns when keyword selection is a hybrid between tagging and expert classification schemes. A keyword analysis conducted by Zhang et al. [26] found that the frequency rank distribution of keywords in the Proceedings of the National Academy of Sciences (PNAS) followed Zipfs law, i.e., *P*_*n*_ ∝ *n*^−*α*^, where *P*_*n*_ is frequency and *n* is rank. This frequency and rank relationship reveals low frequencies of most keywords and high frequency of popular keywords. In addition, the study revealed a power law scaling behavior between cumulative number of keywords and the corresponding cumulative number of distinct keywords indicating universality in scaling [26]. The existence of such scaling relationship was established in several studies related to tagging. Irrespective of the differences in the generation of user-selected tags or creation of academic keywords, they both follow the same scaling law. The study observed an exponential decay of keywords in PNAS, which is similar to that found in other high impact factor journals. They further observed that high impact-factor journals perennially published new and novel topics, while low impact-factor journals continue to publish articles on the same topics and themes for a prolonged period of time. Keyword frequencies alone fail to capture relationships between different keywords. The inability to capture keyword relationships obscures vital information on knowledge components and structure, without which it is not possible to track the evolution of a research field. To address this issue, keyword networks are generally constructed and analyzed using basic network science measures. Such analysis helps one to understand the underlying knowledge structure of a research field. For demonstration of KCN-based approach, we use the case of nanoEHS field.

## 2. Methods

### 2.1 Data collection

Based on previous work [17], the authors investigated literature related to nanoEHS from the Science Citation Index Expanded (SCI-EXPANDED) and Social Sciences Citation Index (SSCI) databases available through the Web of Science. These two databases provide access to more than 8,500 major scientific and technical journals and 3,000 social sciences journals across 200 disciplines. The KCNs are constructed using the Network Workbench software tool [27] to determine the most frequently occurring terms and co-occurrence patterns among them. The search terms include “nano* AND risk analysis”, “nano* AND risk assessment”, “nano* AND risk management”, and “nano* AND risk communication” (here nano* stands for any term starting with nano, e.g., nano manufacturing, nano technology, nano materials, and nano processes). A total of 850 papers were identified. These search results are refined to exclude papers related to areas other than risk analysis as well as those written in other languages. The remaining 627 papers (comprised of journal articles, conference proceedings, reviews, etc.) published between 2000 and 2013 were considered for building KCNs. Given that only four papers were published between 2000 and 2004, those four papers and that time window was excluded from the analysis; the number of papers published is too small to build a meaningful KCN for that period.

The papers published between 2005 and 2013 (623 papers) are separated into three time windows with 3 year durations: 2005–2007, 2008–2010 and 2011–2013. A separate KCN is constructed for each of the three time windows to study temporal evolution of the nanoEHS risk analysis literature between 2005 and 2013. The words and terms provided in the keyword section of the articles were first examined to eliminate redundancy, then further cleaned up data before the construction of KCNs.

### 2.2 Metrics

Co-occurrence networks are most suited to reveal the evolution of a system that has a finite set of entities with non-zero probability of establishing a link between them. The weighted nature of co-occurrence networks calls for network measures that are specific to weighted networks. Applying measures designed for unweighted networks to weighted networks may not yield appropriate results. A set of network measures, designed by Barrat et al. [28] for weighted networks, showed superior representation of the network’s structural characteristics. Duvuru et al. [25] statistically analyzed co-occurrence networks with metrics used for weighted networks to uncover emerging trends in academic research. This work discusses several other relevant network measures typically used for analyzing weighted networks. In general, weighted networks are represented by adjacency matrix *A*_*ij*_ = *a*_*ij*_*w*_*ij*_, where *a*_*ij*_ takes a value of 1 if there exists a link between node *i* and node *j*, otherwise 0. The weights are represented by *w*_*ij*_. The section below reviews the network measures that are relevant to the present work.

#### 2.2.1 Degree

Degree of a node is the total number of links incident on the node. It reflects the relative importance of the node in a network. It is a type of node centrality measure. The degree of node *i* is defined as follows, and in general represented as *k*_*i*_:

$$k_{i}=\sum_{j\in Q_{i}}a_{ij}$$

where *Q*_*i*_ is the set of nodes connected to node *i*.

#### 2.2.2 Strength

For a weighted network, degree of a node may not always be a suitable measure to gauge the node’s relative importance [28]. A weighted network is described by a weighted adjacent matrix *w*_*ij*_, which represents the weight on the link between node *i* and *j*, where *i* = 1, …, *N; j* = 1, …, *N;* and *N* is the number of nodes in the network. Here, only the undirected network with symmetric weights w_*ij*_ = *w*_*ji*_ are considered. The definition of degree can be extended to strength as:

$$s_{i}=\sum_{j\in Q_{i}}w_{ij}$$

where *s*_*i*_ is the strength of node *i*. Strength characterizes importance of a node more accurately than degree since the former is compound measure of both degree and link weights.

#### 2.2.3 Average weight as a function of end point degree

The average weight of a link is defined as follows:

$$<w_{ij}>\sim \left(k_{i}k_{j}\right)$$

where *k*_*i*_ and *k*_*j*_ are degrees of node *i* and *j*, respectively and (*k*_*i*_*k*_*j*_) is their product. This measure allows one to observe co-occurrence of links between pairs of nodes as the degrees of the nodes change.

#### 2.2.4 Average weighted nearest neighbor’s degree as a function of degree

The average weighted nearest-neighbors degree is defined as:

$$k^{w}{}_{nn,i}=\frac{1}{s_{i}}\sum_{j\in Q_{i}}w_{ij}k_{j}$$

The $k^{w}{}_{nn,i}$ is the affinity measure that highlights the tendency of nodes to link with neighbors with similar degree characteristics: high degree nodes link with high degree neighbors and low degree nodes link with low degree neighbors. If the affinity measure is proportional to degree then the network is assortative; if affinity measure is inversely proportional to degree then the network is disassortative [28].

#### 2.2.5 Weighted clustering coefficient as a function of degree

This measure is defined as

$$C_{i}^{w}=\frac{1}{S_{i}\left(k_{i}-1\right)}\sum_{j\in Q_{i},h\in Q_{i}}\frac{\left(w_{ij}+w_{ih}\right)}{2}a_{jh}$$

such that the weighted clustering coefficient acts as a measure of the local cohesiveness, which takes into account the importance of the structure clustered around a node on the basis of the interaction intensity actually found on the local triplets. This is a measure of how cohesive a group of nodes is or how well connected a node is to its neighbors [28].

### 2.3 Chronological analysis

Normally KCNs are constructed covering the entire period of interest, from the nascent stage of the field until the time of analysis. Alternatively, one can divide the lifetime of a field into regular time windows of arbitrary length (e.g., 3- or 4-year time windows), build separate KCNs for each time window, and then comparatively analyze these chronologically ordered KCNS. This approach adds a time dimension to the KCN-based analysis of the scientific literature. It sheds light on the evolution of knowledge components, knowledge structure, and research trends in the field.

### 2.4 Visual analysis

In general, one observes two types of keywords: topical keywords (super set keywords) and specific keywords (subset keywords). For example, “nanomaterial” is considered as a superset and “carbon nanotubes” as a subset. Topical keywords indicate a broad classification of the topics of a field, while specific keywords identify knowledge components and support search and retrieval functions. Statistical analysis on its own reveals macro characteristics, but it is likely to be biased towards topical keywords. This limitation can be overcome by visual analysis, which can give an unbiased view of all keywords. Visual analysis helps researchers identify research directions to advance a scientific field. Statistical analysis in combination with visual analysis provides richer information than any one of them independently.

## 3. Results

The objective of the statistical analysis is to investigate the characterization of nodes, links, and network cohesion in the nanoEHS risk literature. Table 1 shows the summary of statistical analysis for the periods 2005–2007, 2008–2010, and 2011–2013. We observe an approximate doubling of nodes across each time window, which indicates rapid introduction of new keywords resulting in expansion of knowledge structure in the nanoEHS risk literature. In addition, the decrease in average degree and average strength constitutes a key characteristic of knowledge expansion. A reduction in the average degree of nodes indicates the emergence of new nodes (keywords in the field) that have not been previously found in the earlier literature. On the other hand, a reduction in the average strength indicates a reduction in co-occurrence of nodes (keyword pairs in the field). On a macroscopic level, the decreasing average degree and average strength result from the emergence of novel, nascent materials, technologies, and methods in the field. These low-degree and low-strength nodes (keywords in the field) are potential candidates for further investigation for scientists and engineers. At a microscopic level, researchers concerned with nanoEHS risk can either focus on high-degree or high-strength nodes, which represent established materials, technologies, and methods.

**Table 1:** Topological characteristics of KCNs for nanoEHS literature for three time windows: 2005–2007, 2008–2010, and 2011–2013.

	2005–2007	2008–2010	2011–2013
**Number of Articles**	59	215	349
**Nodes**	208	565	912
**Links**	1049	2438	3252
**Average Strength <s>**	10.47	9.44	7.48
**Max Strength**	100	247	292
**Average Degree <k>**	10.09	8.63	7.13
**Max Degree**	79	173	239
**Average Weight <w>**	1.04	1.09	1.05
**Max Weight**	6	18	18

**Fig 2:**
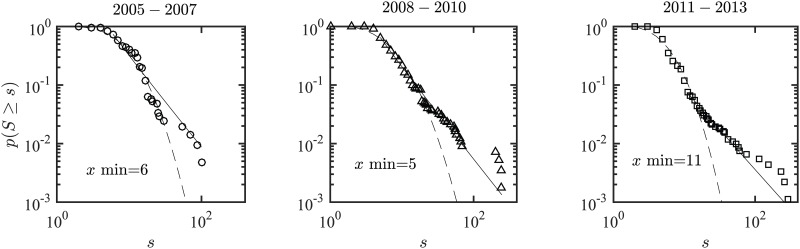
Complementary Cumulative Distribution Function (CCDF) of the keyword co-occurrence network. The solid line represents a power law fit and the dashed line represents a lognormal fit. The *xmin* value in each figure represents a minimum value from where the power law fit is best approximated (*xmin* = 6 for 2005–2007, *xmin* = 5 for 2008–2010 and *xmin* = 11 for 2011–2013). The strength distribution for the year 2005–2007 can be approximated by a lognormal distribution (*μ* = 1.849, *σ* = 0.7952385), while the strength distribution for the years 2008–2010 and 2011–2013 can be approximated by power law. The scaling exponent parameter for the power law approximation are *α* = 2.469 for the year 2008–2010 and *α* = 2.348 for the year 2011–2013. The *x* and *y* axis are in logarithmic scale.

Fig 2 shows the strength distribution of the KCN for periods 2005–2007, 2008–2010, and 2011–2013. The *y* axis in Fig 2 represents the complementary cumulative distribution function (CCDF) and the *x* axis represents the strength values. Both axes use logarithmic scale. The power law distribution fit is determined for the region greater than *xmin*, the value of which is determined by a Kolmogorov–Smirnov test. For estimated *xmin*, the scaling parameter *α* is approximated using the maximum likelihood estimation method. For a set of estimated *xmin* and *α*, the power law hypotheses is tested using the bootstrap method; the *p-value*s are computed to accept or reject the possibility of the underlying distribution to be a power law approximation. The strength distribution for keyword co-occurrence network can be approximated by a lognormal distribution (*μ* = 1.849, *σ* = 0.7952385) for time period 2005–2007 and by a power law distribution for time periods 2008–2010 and 2011–2013. The scaling exponent parameter for the power law approximation are *α* = 2.469 for the year 2008–2010 with *p*-value 0.213 and *α* = 2.348 for the year 2011–2013 with *p*-value of 0.0.85. A *p-value* > 0.1 indicates the plausibility of a power law. S1 Supporting Information summarizes the fitting procedure, parameter estimation and goodness of fit values for all time periods. For a detailed procedure for fitting power law distribution to data one can refer to the widely cited work of Clauset et al. [29].

**Fig 3:**
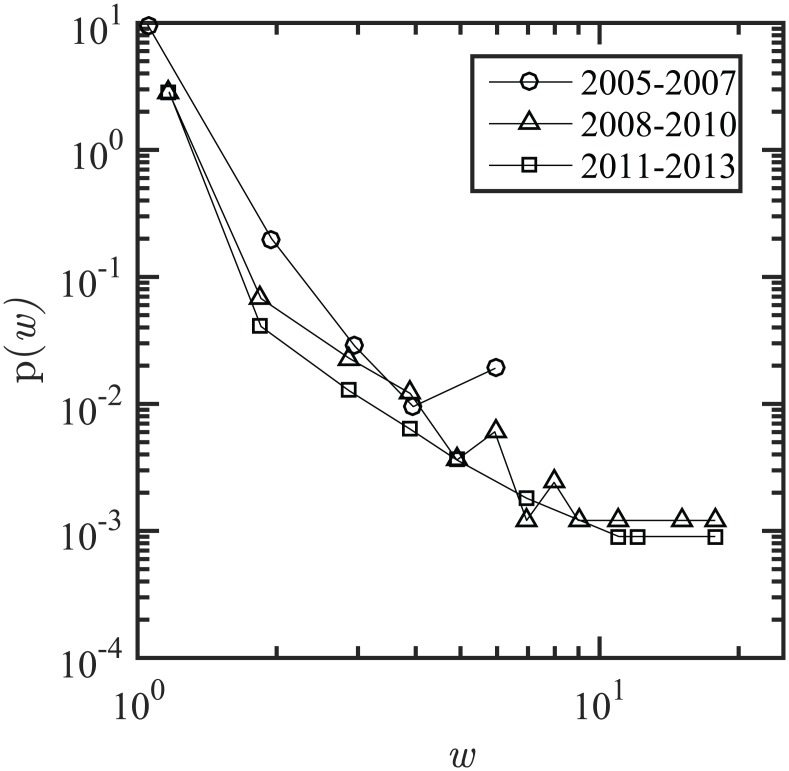
Weight distribution of the keyword co-occurrence networks for three time periods. First period 2005–2007, second period 2008–2010, third period 2011–2013; the *x* and *y* axis are in logarithmic scale.

A shift in strength distribution from lognormal to power law is observed, indicating that the network topologies subsequent to 2005–2007 are scale-free networks, with high heterogeneity (i.e., fewer nodes with high strength values and a higher number of nodes with lower strength values; it translates to fewer keywords with a larger numbers of co-occurrence, instead of many keywords with smaller counts of co-occurrences). In addition, a decaying pattern for weight distribution is observed for all three time periods, which indicates a lower frequency of links with large weights and higher frequency of links with small weights. Fig 3 shows the PDF of weight distribution for all three time periods.

**Fig 4:**
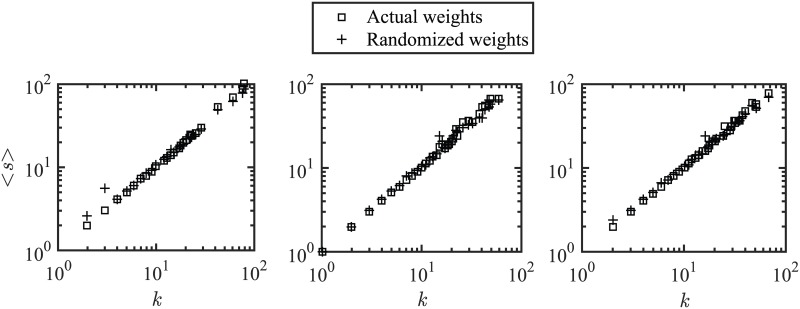
Strength vs. node degree of the keyword co-occurrence networks for three time periods. First period 2005–2007 (left figure), second period 2008–2010 (middle figure), third period 2011–2013 (right figure); the *x* and *y* axis are in logarithmic scale.

Fig 4 illustrates the average strength as a function of degree for actual KCN and random network (created by distributing the weights of the actual network randomly). To determine whether the link weights are random, the average strength as a function of degree is compared for actual KCN and random networks [28]. The average strength relationship with the degree can be captured using the scaling relation, *s*(*k*) ~ *k*^*α*^. We observe *α* = 1 for all three time periods (2005–2007, 2008–2010, 2011–2013), indicating that both entities (strength and degree) provide the same information about the keyword co-occurrence system. A value of *α* > 1 would indicate that the strength of a node grows faster than its degree.

**Fig 5:**
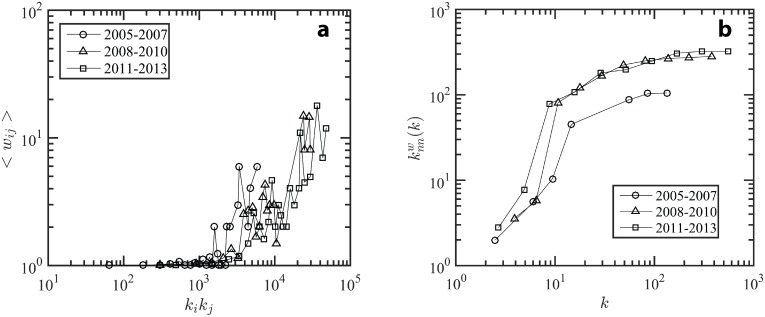
The trends in the keyword co-occurrence network parameters. (a) Average weight vs. endpoint degree and (b) average weighted nearest neighbor degree vs. node degree for three time periods: 2005–2007, 2008–2010, 2011–2013.

The strength distribution and the strength vs. degree relation constitute node properties while the weight distribution constitute link properties. However these metrics alone are insufficient to measure the relationships between nodes. For assessing the relationship between different nodes, the authors use 1) average weight vs. endpoint degree, 2) average weighted nearest neighbor degree vs. degree, and 3) weighted clustering coefficient vs. degree. The endpoint degree is calculated by multiplying the degrees of the nodes on each end of the link (*k*_*i*_*k*_*j*_). Fig 5a shows that the average weight in the keyword network for each period increases sharply for values of *k*_*i*_*k*_*j*_ > 10^3^. It indicates that the tendency of co-occurrence increases sharply for high degree keywords. However, one cannot be sure whether high degree nodes (keywords) pair up with high degree nodes or low degree nodes (i.e., several combinations of node degrees can result in the same values for *k*_*i*_*k*_*j*_, e.g., *k*_*i*_*k*_*j*_ = 15*1 or *k*_*i*_*k*_*j*_ = 3*5). This issue is overcome by analyzing average weighted nearest neighbor degree (see Fig 5b). Using this measure, one can ascertain the assortative behavior of the KCN. Fig 5b shows an increase in average weighted nearest neighbor degree with increase in node degree, revealing the assortative behavior of the network (i.e., high degree keywords tend to link up with high degree keywords, while the low degree keywords tend to link up with low degree keywords). However, assortative behavior is not uniformly observed across all degrees. The value of average weighted nearest neighbor degree increases rapidly between degree 2 to degree 10; thereafter, the rate of increase declines and reaches a plateau for degrees greater than 80. This indicates absence of topological correlations for high degree keywords. This implies that the nanoEHS researchers are developing or experimenting with new methods and nanomaterials, indicating a desirable trend. To explore whether high degree nodes connect to low degree nodes, the average weighted clustering coefficient was utilized. The relationship between the average weighted cluster and degree determines whether the keywords form cohesive groups or clusters in the keyword co-occurrence system.

**Fig 6:**
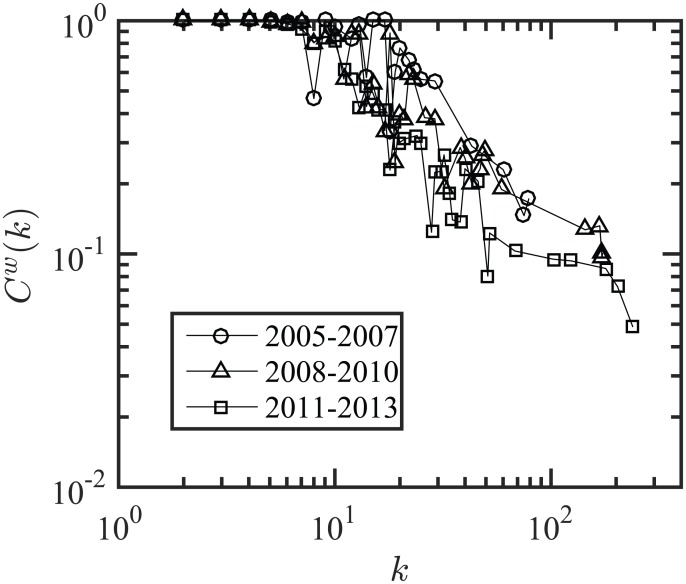
Weighted clustering coefficient vs. degree for three time periods. First period 2005–2007, second period 2008–2010, third period 2011–2013; the nodes with degree around 10 form clusters, whereas the nodes with degree around 100 do not form clusters, i.e., keywords with small degrees form connected communities of words, but keywords with large degrees connect with isolated keywords.

Fig 6 shows that keywords with a smaller degree form clusters with other smaller degree keywords, whereas keywords with a large degree connect to many keywords, and do not form clusters. In other words, hub-keywords are connected to a large set of keywords, but the members of the set themselves co-occur less frequently.

In summary, the KCNs for nanoEHS show an increase in average weight with endpoint degree, indicating the co-occurrence of keyword pairs. The average weighted nearest neighbor degree shows the tendency of low degree nodes to attach with other low degree keywords (i.e., assortative for low degree keywords) while the high degree keywords exhibit disassortative behavior. Finally, the average weighted clustering coefficient indicates the link between a high degree keyword and a low degree keyword. The aforementioned metrics are useful to uncover macro trends pertaining to scientific trends if a keyword convention of collaborative tagging and classification are followed together. If the keyword system is based on random user based tagging alone, the strength distribution follows a Poisson distribution. Average weight as a function of endpoint degree showed no relationship, and average weighted clustering coefficient that differs from that of a scale free network failed to capture the scientific trends [25].

Table 2 displays the top twenty keywords by strength for years 2005–2007, 2008–2010, and 2011–2013. We can see the evolution of the keywords across the temporal frames. However as mentioned earlier, academic keyword article selection process is a combination of classification and tagging; this makes a difference in interpretation of what the keywords mean. From Table 2 one can see that the two keywords, nanomaterial and nanoparticle, seem to be redundant but nanoparticle is a subset of nanomaterial.

**Table 2:** Top twenty keywords by strength.

Years 2011–2013		Years 2008–2010		Years 2005–2007	
Node	Strength	Node	Strength	Node	Strength
Nanoparticle	292	Risk Assessment	247	Risk Assessment	100
Risk Assessment	260	Nanotechnology	246	Nanotechnology	87
Nanomaterial	233	Nanoparticle	225	Nanoparticle	69
Nanotechnology	152	Nanomaterial	199	Nanomaterial	54
Nanotoxicology	116	Exposure	69	Fullerenes	30
Toxicity	77	Toxicity	66	Pulmonary Toxicity	26
Risk Management	61	Carbon nanotube	64	Toxicity	25
Engineered Nanomaterial	58	EHS (Environmental, Health, and Safety)	57	Environment	24
Silver Nanoparticle	53	Nanotoxicology	56	Dermal Toxicity	24
Titanium Dioxide	48	Multi-wall Carbon Nanotube	55	Genotoxicity	24
Nanotoxicity	42	Risk Management	53	Carbon nanotube	21
Carbon nanotube	37	Risk	48	Ultrafine Particles	20
Risk	37	Titanium Dioxide	42	Occupational Health	18
Genotoxicity	37	Regulation	39	Nanostructured Materials	17
Oxidative Stress	36	Exposure Assessment	38	Quantum Dots	17
Environment	32	Environment	36	Nanowires	17
Silver	31	Toxicology	36	Nanorod	17
Gold Nanoparticle	28	Health	33	Nanocluster	17
Nanosilver	25	Cytotoxicity	32	Nanocrystal	17

**Fig 7:**
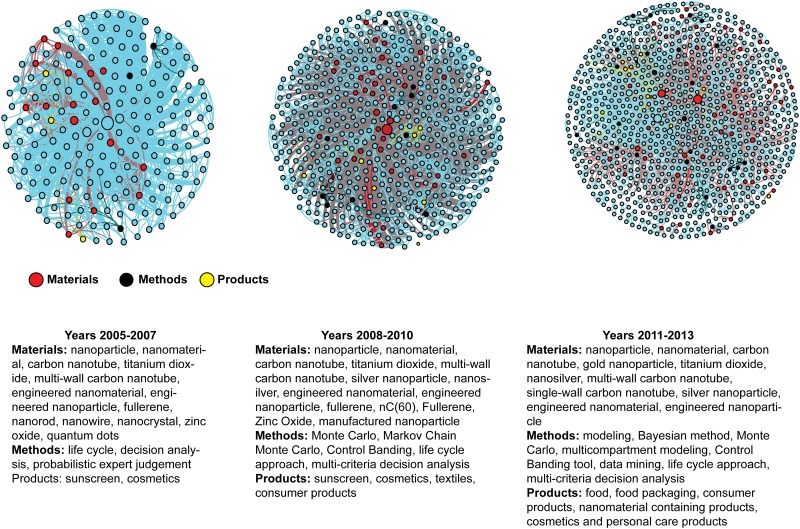
Visual representation of the KCNs for three time periods along with important keywords. The three periods for keyword evolution are 2005–2007, 2008–2010, and 2011–2013 respectively.

Three KCNs, one for each of the three time periods (2005–2007, 2008–2010, and 2011–2013), are shown in Fig 7. They map the evolution of materials, products, and methodologies in connection with nanoEHS risk analysis.

Between 2005 and 2007, hazard and toxicity risk for various types of nanoscale materials including nanoparticles, such as quantum dots, fullerenes, carbon nanotubes (e.g., single-wall carbon nanotubes, multi-wall carbon nanotubes) and nano-titanium dioxide, were the major materials investigated. In addition, different forms of nanomaterials such as nanorods, nanowires, nanopowders, and nanocrystals were also studied. In addition to characterization of the materials, the health effects of nano-enabled products such as nanomedicines, sunscreen and cosmetics were also explored. Methodologies such as data mining, probabilistic expert judgement, decision analysis and life cycle approaches were common in analysis of the EHS risk of nanomaterials between the years 2005 and 2007. During 2008–2010 time period, the toxicity of silver nanoparticle and nano-silver were studied in addition to carbon based materials such as CNTs, carbon black, bucky-balls (nC(60)). Textile products began to be investigated in addition to sunscreen and cosmetics. Monte Carlo and Markov Chain Monte Carlo simulation methods became popular techniques to study the EHS risk of nanomaterials between 2008 and 2010. Control banding and multi-criteria decision analysis methods as risk management techniques emerged as common tools to reduce the EHS risk of nanomaterials.

For the final time period between 2011 and 2013, in addition to carbon-based nanomaterials, nano-silver, and nano-titanium dioxide, gold nanoparticles were studied for the first time. Furthermore, the effect of food, food packaging, and personal care products containing nanomaterials on human health became a popular research topic. As a methodology, the Bayesian method was applied for the first time in the literature to analyze the nanoEHS risk between 2011 and 2013. Moreover, a multi-compartment modeling technique was used to analyze the EHS risk of nanomaterials during their different stages. Other modeling techniques such as non-linear and chance constraint programming approaches were also applied to make decisions under the conditions of uncertainty in EHS risk of nanomaterials. To summarize, the visual analysis clearly shows adoption of diverse methods for nanoEHS research and investigation of a variety of more nanomaterials. The focus on products show a shift from cosmetics to food and consumer products.

## 4. Discussion

Since 2005, there has been a rapid expansion of knowledge structure in nanoEHS risk literature (see S2 Supporting Information). The number of keywords approximately doubled every three years. The distribution of the number of keyword co-occurrences shifted from a lognormal to power law, i.e., subsequent to 2005–2007, fewer keywords with more co-occurrence, more keywords with small count of co-occurrences. Over the years, the frequency of co-occurrences has grown faster than the growth of number of keywords. The keywords exhibit assortative behavior, i.e., high degree keywords tend to link up with high degree keywords while the low degree keywords tend to link up with low degree keywords. This assortative behavior is more pronounced for keywords that link to 10 or fewer keywords. This indicates that the nanoEHS community has been engaged in developing or experimenting with new methods and nanomaterials. Keywords with smaller degrees form clusters with smaller degree keywords whereas keywords with large degrees connect to the keywords that do not form clusters among them, i.e., keywords appear frequently in the articles with the keywords that appear rarely in the articles together.

Between 2005 and 2007, the hazard and toxicity risk of various types of nanoscale materials, new forms of nanomaterials, as well as the health effects of nano-enabled products were investigated. During this period new methodologies such as data mining, probabilistic expert judgement, decision analysis and life cycle approaches were applied for EHS risk analysis.

During the 2008–2010 time period, the toxicity of silver nanoparticles, CNTs, carbon black, bucky-balls (nC(60), nano-enabled textile products, sunscreen and cosmetics were studied more actively. During this same time frame, Monte Carlo simulation, control banding and multi-criteria decision analysis methods became popular techniques to study the EHS risk of nanomaterials.

More recently, between 2011 and 2013, the EHS risk of gold nanoparticles was studied. The effect of nanomaterial-based food, food packaging, and personal care products on human health became an active research topic. Bayesian and multi-compartment modeling techniques were employed to analyze the EHS risk of nanomaterials. Other modeling techniques such as non-linear and chance constraint programming approaches were also applied to make decisions under the conditions of uncertainty in EHS risk of nanomaterials.

## 5. Conclusion

In this paper, keyword co-occurrence networks are used to reveal insights into knowledge structures and their temporal dynamics of an evolving research field such as nanoEHS risk assessment. This work introduces novel analysis techniques relevant to weighted networks other than network metrics such as betweenness centrality and modularity to gain a deeper understanding of the knowledge structures. The combination of statistical analysis to uncover macro trends and visual analysis to observe micro trends serve as an effective approach to analyze trends and patterns in a literature of an emerging research field. The statistical analysis is particularly useful when the keyword system follows a combination of expert classification and collaborative tagging as opposed to random user based tagging alone.

Systematic literature reviews often focus on the results and methodologies that are presented in individual studies, and can result in detailed qualitative mapping of the body of research work. If the objective of a literature review is only to gain a macro level understanding of research subject, e.g., introduction of novel methodologies or evolution of traditional methodologies, then an in-depth comprehensive systematic literature review is time consuming. The KCN-based analysis, requiring far less time, enables macro level quantitative mapping that reveal temporal evolution of the research subject. Unlike the traditional systematic literature reviews, the KCN-based analysis will also shed light on the connections between keywords, key concepts, and key methods and methodologies through statistical measures. In the present nanoEHS KCN-based analysis, the findings on new methods, materials of interest, and product applications are aligned with what were observed through a traditional detailed literature review [17]. This observation supports the concept that KCN-based analysis can be conducted quickly to explore a vast amount of literature prior to undertaking a rigorous time-consuming systematic review. The proposed pre-systematic-review analysis can provide a structured map to conducting a literature search, as well as significantly reduce the effort required for a systematic review.

The present work demonstrates the effectiveness and usefulness of the KCN-based analysis to discover knowledge components and knowledge structure of the nanoEHS risk assessment field, however, the proposed methodology and techniques can be readily applied to any other scientific literature.

## Supporting information

S1 Supporting Information Estimation of lower bound xmin and scaling parameter *α*. (DOCX)

S2 Supporting Information Nano Environmental, Health, and Safety (NanoEHS) risk literature. (DOCX)
