# Correction: Novel keyword co-occurrence network-based methods to foster systematic reviews of scientific literature

**DOI:** 10.1371/journal.pone.0185771

**Published:** 2017-09-27

**Authors:** Srinivasan Radhakrishnan, Serkan Erbis, Jacqueline A. Isaacs, Sagar Kamarthi

There is an error in the eighth sentence of the second paragraph of the Results section. The correct sentence is: The scaling exponent parameter for the power law approximation are *α* = 2.469 for the year 2008–2010 with *p*-value 0.213 and *α* = 2.348 for the year 2011–2013 with *p*-value of 0.85.

## References

[1] Radhakrishnan S, Erbis S, Isaacs JA, Kamarthi S (2017) Novel keyword co-occurrence network-based methods to foster systematic reviews of scientific literature. PLoS ONE 12(3): e0172778 https://doi.org/10.1371/journal.pone.0172778 28328983 10.1371/journal.pone.0172778 PMC5362196

