# Supplementary material for: Novel keyword co-occurrence network-based methods to foster systematic reviews of scientific literature
Source: PLoS One. 2017 Mar 22;12(3):e0172778. doi: 10.1371/journal.pone.0172778 (PMC5362196; doi:10.1371/journal.pone.0172778)
Supplement: S1 Supporting Information — (DOCX) [file pone.0172778.s001.docx]

**S1 Supporting Information**

**Estimation of lower bound *xmin* and scaling parameter *α***

In this work we use the method described by Clauset et al [35] to determine the best distributions that represent the data.

In general, if an empirical distribution is approximated using power law distribution, it is so for values of *x* greater than *xmin* (lower bound). In order to calculate the scaling parameter *α* , the values of *x*  below the lower bound are discarded. The value of lower bound must be carefully chosen, because a high value of a lower bound will discard many points below the lower bound, which in turn increases statistical error on the scaling parameter and bias from finite size effect. On the other hand, a low value for the lower bound will yield a biased scaling parameter, because there is an attempt to fit a power law model to non-power law data [35].

In order to find *xmin*, an iterative process is used where each data point is considered as *xmin*. For every data point considered as *xmin*, the values of *x*< *xmin* are truncated. Then all values of *x*> *xmin* including *xmin* are used to compute the empirical CCDF. The theoretical CCDF is calculated using *p*(*x*) = (*x*/*xmin*)^(-^*^α^*^+1)^ where the value of *α* is calculated by the maximum likelihood estimator (MLE) method. The maximum absolute difference between the empirical CCDF and theoretical CCDF is computed as the Kolmogorov-Smirnoff (KS) statistic. The aforementioned steps are repeated for each data point and the *xmin* corresponding to the minimum value of KS statistic is considered as the true *xmin*. After the true *xmin* is computed, the scaling parameter *α* corresponding to the true *xmin* is computed using MLE method.

**Goodness of Fit**

Having the estimated lower bound and scaling parameter alone will not ensure the applicability of power law distribution. Hence, we use a bootstrapping method to confirm the hypothesis that the empirical data distribution can be approximated by a power law distribution. In this method, a large number of synthetic data sets are generated using the parameters estimated using the aforementioned technique. Each synthetic data is then fit to its own power law model, and the KS statistic is computed for data relative to its own model. Then we count for what fraction of time the resulting KS statistic value is larger than the value for empirical model. This fraction is represented by the *p-value*. It is important to note that each time the KS statistic is computed between the synthetic data and its respective model (and not the empirical data). This ensures an unbiased estimate of *p-value*. For further details on how to generate the synthetic data refer to Clauset et al [35]. A significance level of 0.1 is used, which means that a *p-value* greater than 0.1 fails to reject the null hypothesis (note that the null hypothesis is that the empirical distribution is drawn from power law distribution). A *p-value* less than 0.1 rules out the possibility of power law distribution.

**Ruling out Alternate Distributions**

Having a *p-value* greater than 0.1 does not necessarily rule out possibility of alternate distributions. There may arise a case where alternate distributions like lognormal and exponential distribution will also yield a *p-value* greater than 0.1. In such cases the power law distribution is compared to alternate distributions via likelihood ratio test. The basic idea behind the likelihood ratio test is to compute the likelihood of the data under two competing distributions. The one with the higher likelihood is then the better fit. Alternatively one can calculate the ratio of the two likelihoods, or equivalently the logarithm $\mathcal{R}$ of the ratio, which is positive or negative depending on which distribution is better, or zero in the event of a tie [35]. The dependence on the sign of the log likelihood ratio alone will not indicate which model is the better due to the presence of statistical fluctuation. To ensure that the observed value of $\mathcal{R}$ is sufficiently far from zero, the standard deviation σ on $\mathcal{R}$ [35] must be known. This is estimated using a method proposed by Vuong [36]. The method gives a *p*-value that tells us whether the observed sign of $\mathcal{R}$ is statistically significant [35]. If this p-value is small (say *p* < 0.1) then it is unlikely that the observed sign is a chance result of fluctuations and the sign is a reliable indicator of which model is the better fit to the data. If *p* value is large on the other hand, the sign is not reliable and the test does not favor either model over the other [35]. In this case the null hypothesis is formed by an assumption that both distributions are far from the true distributions and alternate hypothesis is formed by an assumption that one of the distribution is closer to the true distribution. The other alternative to likelihood ratio test are Bayesian approaches, Cross validation and Minimum description length (MDL)

**Results for Keyword Co-occurrence Network**

The strength distribution for period 2005-2007 can be better approximated using a lognormal distribution as opposed to a power law distribution. The bootstrapping method yielded a *p*-value of 0 for power law distribution and yielded a value of 0.2 (*p-*value >0.1) for the lognormal distribution.

The strength distribution for period 2008-2010 can be better approximated using a power law distribution. The bootstrapping method yielded a *p*-value of 0.213 (*p-*value >0.1) for the power law distribution and yielded a value of 0.09 for a lognormal distribution. The Vuong test statistic yielded a positive 0.3 value favoring power law over lognormal with *p*-value 0.08 (*p-*value < 0.1)

The strength distribution for period 2011-2013 can be better approximated using a power law distribution. The bootstrapping method yielded a *p*-value of 0.85 (*p-*value >0.1) for the power law distribution and yielded a value of 0.4 (*p-*value >0.1) for a lognormal distribution. The Vuong test statistic yielded a positive 0.612 value favoring power law over lognormal with *p*-value 0.09 (*p-* value < 0.1)

| Period | Distribution Approximation | Parameters |
| --- | --- | --- |
| 2005-2007 | Lognormal | *μ* = 1.849, *σ* = 0.7952385 |
| 2008-2010 | Power law | *xmin* = 5, *α* = 2.469 |
| 2011-2013 | Power law | *xmin* = 11, *α* = 2.348 |
