# Supplementary material for: Novel keyword co-occurrence network-based methods to foster systematic reviews of scientific literature
Source: PLoS One. 2017 Mar 22;12(3):e0172778. doi: 10.1371/journal.pone.0172778 (PMC5362196; doi:10.1371/journal.pone.0172778)
Supplement: S2 Supporting Information — (DOCX) [file pone.0172778.s002.docx]

**S2 Supporting Information**

**Nano Environmental, Health, and Safety (NanoEHS) Risk Literature**

The literature in the nanoEHS field has been advancing over the years since the enactment of the U.S. Nanotechnology Initiative (NNI) in 2001. Since then, the cumulative U.S. investment is nearing $24 billion though fiscal year 2017, with investments in the environmental health and safety research associated with various nano-related technologies nearing $1 billion [1]. With a goal to support responsible development of nanotechnology, the NNI agencies have promoted research to identify potential risks as well as methods to assess and manage the risks. As part of the U.S 2014 NNI Strategic Plan, nanoEHS involves multidisciplinary research that is focused in six interrelated areas: Nanomaterial Measurement Infrastructure; Human Exposure Assessment; Human Health; Environment; Risk Assessment and Risk Management Methods; and Informatics and Modeling [2, 3]. One objective of nanoEHS research lies in strengthening the scientific foundation of risk assessment and risk management methods for predictive use. Predictive models on hazards are desirable, because the number of toxicological studies for nanoparticle hazard evaluation that would need to be undertaken to fully assess the effects on human health and the environment is estimated to range from $249 million for screening assays to $1.18 billion for long-term in vivo testing for all nanomaterials [4], requiring 34-53 years for complete testing. Hence various forms of risk analysis, including risk assessment, risk management, and risk communication, can provide more timely and cost effective approaches for managing and regulating potential hazards of nanomaterials. With the number of nano-enabled products rising in energy, electronics, medicine, automotive, and other consumer applications – some already commercialized and in the market [5], the use of nanoEHS risk analysis techniques has been steadily growing to assess and mitigate potential hazards. Challenges to developing comprehensive risk management policies include the diversity of nanomaterials and lack of systematic and standardized risk assessment methodologies [6]. Over the past 15 years, the literature published in this area has evolved, providing important insights on risk assessment and risk management tools associated with nanotechnology, nanomanufacturing, and nano-enabled products.

**Reference**

1. U.S. National Nanotechnology Initiative (NNI), (2016) NNI Supplement to the President’s Budget for Fiscal Year 2017, Subcommittee on Nanoscale Science, Engineering, and Technology Committee on Technology National Science and Technology Council, March 2016.

2. U.S. National Nanotechnology Initiative (NNI), (2014) 2014 NNI Strategic Plan, February 2014.

3. U.S. National Nanotechnology Initiative (NNI), (2014) Progress Review on the Coordinated Implementation of the National Nanotechnology Initiative 2011 Environmental Health and Safety Research Strategy, June 2014

4. Choi JY, Ramachandran G, Kandlikar M. The impact of toxicity testing costs on nanomaterial regulation. Environmental science & technology. 2009 Feb 20;43(9):3030-4.

5. The Project on Emerging Nanotechnology, Consumer Product Inventory, http://www.nanotechproject.org/cpi/ Last accessed, December 15, 2016.

6. Lubick N. Risks of nanotechnology remain uncertain. Environmental science & technology. 2008 Mar 15;42(6):1821-4.
